# Prior Evaluation of Nutritional Status and Mortality in Patients with Sepsis in South Korea

**DOI:** 10.3390/nu15245040

**Published:** 2023-12-08

**Authors:** Tak Kyu Oh, In-Ae Song

**Affiliations:** 1 Department of Anesthesiology and Pain Medicine, Seoul National University Bundang Hospital, Seongnam 03080, Republic of Korea; 66034@snubh.org; 2 Department of Anesthesiology and Pain Medicine, College of Medicine, Seoul National University, Seoul 01811, Republic of Korea

**Keywords:** sepsis, septic shock, critical care, intensive care unit, body mass index

## Abstract

Our objective was to determine whether nutritional status correlates with mortality in sepsis patients. Data from a nationwide registration database were utilized for this population-based cohort study. The study subjects comprised adults who received standard health examinations before being admitted to the hospital for sepsis and were diagnosed with sepsis between 2018 and 2020. Nutrition scores were evaluated using the Nutritional Lifestyle Assessment Tool in South Korea. Overall, 2482 patients with sepsis were included in this study. The 90-day and 1-year mortality rates in patients with sepsis were 26.8% (664/2482) and 34.2% (848/2482), respectively. In the covariate-adjusted multivariable logistic regression model, a 1-point increase in nutrition score was associated with a decrease in 90-day mortality in patients with sepsis (odds ratio [OR]: 0.98, 95% confidence interval [CI]: 0.97, 0.98; *p* = 0.025). In the covariate-adjusted multivariable Cox regression model, a 1-point increase in nutrition score was associated with a decrease in 1-year mortality in patients with sepsis (hazard ratio [H.R.]: 0.99, 95% CI: 0.98, 0.99; *p* = 0.035). Our results suggest that adequate dietary intake and healthy eating habits might protect against mortality among sepsis patients.

## 1. Introduction

Sepsis is one of the leading causes of death owing to the malfunction of life-supporting organs [1]. According to a report on the global burden of disease for 2017, about 48.9 million cases of sepsis and 11.0 million fatalities related with sepsis were documented around the globe [2]. In order to combat such a significant threat to public health, it is necessary to develop new preventative measures and make progress in treating sepsis [3].

There is ongoing research on the factors that determine a patient’s prognosis when they have sepsis [4]. Due to the possible stimulation of an acute catabolic response by this condition, which may result in the depletion of energy stores within the organism, the provision of sufficient nutritional support is a crucial and disputed matter among patients who are afflicted with sepsis [5,6]. Therefore, nutritional support is essential in the management of critically sick patients in order to expedite the patients’ recovery and bring down their rates of morbidity and death [7]. Patients who are critically ill and are being treated in intensive care units (ICUs) have a higher risk of passing away if they are malnourished [8]. In addition, malnutrition is a risk factor in and of itself for poor survival outcomes in patients who are hospitalized with sepsis [9,10]. On the other hand, there are not enough techniques available to evaluate the nutritional state of individuals who have sepsis, which is important because it may be associated with death.

In South Korea, nutrition scores produced from questionnaires are considered a nutritional lifestyle evaluation tool. These scores are utilized in standard health examinations that measure the nutritional status of adults, and they are used to evaluate the individuals’ diets. The nutritional status of an individual is determined using scores that represent the individual’s nutritional lifestyle as part of a normal health examination in South Korea.

We intend to investigate the association between nutritional status and mortality among sepsis patients using their nutrition scores from the Nutritional Lifestyle Assessment Tool. We hypothesize that malnutrition caused by low nutrition scores in sepsis patients leads to poor survival outcomes.

## 2. Materials and Methods

### 2.1. Study Design, Ethical Statement, and Data Source

This was a population-based cohort study. The Institutional Review Board of Seoul National University Bundang Hospital approved the study protocol (approval date: 5 January 2022, approval number: X-2201-735-902; study title: Patients with sepsis in South Korea). The Big Data Center of the National Health Insurance Service (NHIS) (NHIS-2022-1-337) approved the study protocol for data sharing. The requirement for informed consent was waived because data analyses were performed retrospectively using anonymized data derived from the South Korean NHIS database. This study followed the ethical standards of the Responsible Committee on Human Experimentation (institutional) and the Helsinki Declaration of 1975.

As South Korea’s only publicly funded health insurance program, the National Health Insurance Service (NHIS) maintains a database with information pertaining to disease diagnosis that is coded according to the International Statistical Classification of Diseases and Related Health Problems, 10th Revision (ICD-10). In addition, in order for the government to be responsible for covering the cost of treatment, prescribing information for medications and procedures need to be registered in the NHIS database by medical professionals.

South Korea has a single public insurance system, the NHIS, and each subscriber pays a varied premium based on their income or assets. Despite paying various insurance premiums, all subscribers can obtain the same governmental medical service assistance benefits. This may help to lessen the selection bias in our research of patients with sepsis.

### 2.2. Study Population

Initially, we screened patients admitted to the hospital with a diagnosis of sepsis (ICD-10 codes: A40, A41, and R65.2) between 1 January 2018 and 31 December 2020. We excluded patients with multiple (two or more) hospital admissions associated with sepsis to focus on the last hospital admission. We excluded patients with sepsis who did not undergo standard health examinations before hospital admission or lacked data regarding nutrition scores.

In South Korea, NHIS subscribers over the age of 40 are advised to have standardized health checkups every two years, and the government pays for these examinations [11]. As a result, 50% of all adults aged 40 years and older may be advised to have routine health checkups in a specific year (i.e., the year preceding sepsis). Furthermore, a recent study found that 70–80% of South Koreans participated in standardized health examinations; however, this varied depending on age, handicap, and socioeconomic position [12].

### 2.3. Nutrition Score Using a Nutritional Lifestyle Assessment Tool

In standard health examinations, a questionnaire called the Nutritional Lifestyle Assessment Tool was used to evaluate the nutritional status of the adults (Table 1). Based on the responses to the questionnaire, we calculated the nutrition scores of patients with sepsis, ranging from 0 to 55 points, before hospital admission.

### 2.4. Study Endpoint

Two endpoints were established in this study. The primary endpoint was 90-day mortality, defined as death within 90 days of hospital admission. The secondary endpoint was 1-year mortality, defined as death within one year of hospital admission.

### 2.5. Covariates

Data regarding age and sex were collected as demographic information. Data regarding the main diagnosis of sepsis were also collected. The main diagnosis was defined as the disease for which the patient’s demand for treatment or examination was the greatest during hospitalization. For example, for a patient who was initially admitted with a diagnosis of urinary tract infection and diagnosed with sepsis during treatment, the main diagnosis would be sepsis if the NHIS determined that the patient’s demand for treatment or examination for sepsis was the greatest. Data regarding residence and employment status were collected as socioeconomic-status-related information. Body mass indices (BMIs, kg/m^2^) at the time of sepsis diagnosis were collected and classified into four groups: <18.5 kg/m^2^ (underweight), 18.5–24.9 kg/m^2^ (normal), 25.0–29.9 kg/m^2^ (overweight), and >30.0 kg/m^2^ (obese). Considering the comorbidity status of patients with sepsis, the Charlson comorbidity index (CCI) and disability information were collected. The CCI scores were calculated using registered ICD-10 codes for individual diseases before hospital admission, as shown in Table S1. In South Korea, all individuals with disabilities are registered in the NHIS database to receive social welfare benefits from the government. Each disability was diagnosed and determined strictly according to the laws of specialist doctors in each field. Difficulty in maintaining daily life was an important criterion for determining disability. Each patient was assigned one of six grades according to severity (1st: most severe; 6th: mildest). In this study, the 1st–3rd grades of disability were considered “severe”; the 4th–6th grades were considered “mild to moderate”. Data regarding ICU admission, mechanical ventilatory support, extracorporeal membrane oxygenation support, and continuous renal replacement therapy use were collected. The patients were classified into three groups according to the hospitals to which they were admitted for sepsis: general hospitals, hospitals, and long-term facility care hospitals. Admission to the internal medicine department and surgery-associated hospital admissions were included as covariates. Data on the length of hospitalization (days) and the total cost of hospitalization associated with sepsis (dollars) were also collected.

### 2.6. Statistical Analysis

Median values with interquartile range (IQR) and ranges for continuous variables and percentages for categorical variables were used to present the clinicopathological characteristics of the patients. First, the log odds of 90-day mortality and the log relative hazard of 1-year mortality according to nutrition scores were presented using restricted cubic splines (RCSs). For 90-day mortality, we performed univariable and multivariable logistic regression analyses, and all covariates were included in the multivariable model. The results are presented as odds ratios (ORs) with 95% confidence intervals (CIs). Hosmer–Lemeshow statistical testing was used to confirm that the goodness of fit in the model was appropriate. For 1-year mortality, univariable and multivariable logistic regression analyses were performed as time-to-event analyses, and all covariates were included for multivariable adjustment in the multivariable model. Specifically, age, sex, main diagnosis of sepsis, residence at sepsis (urban area or rural area), BMI, CCI, disability (mild to moderate or severe), ICU admission, mechanical ventilatory support, extracorporeal membrane oxygenation support, continuous renal replacement therapy use, type of hospital, admitting department (IM or non-IM), surgery associated admission, and year of hospital admission were collected as covariates for multivariable adjustment. A log-log plot was used to confirm that the central assumption of the Cox proportional hazards model was satisfied. Subgroup analyses according to BMI were performed in patients with sepsis because BMI is generally used to evaluate the nutritional status of critically ill patients [13]. No multicollinearity issues were noted among the variables, with a variance inflation factor criterion of <2.0. All statistical analyses were performed using R software (version 4.0.3, R packages, R Project for Statistical Computing, Vienna, Austria). *p* < 0.05 was considered statistically significant.

## 3. Results

### 3.1. Study Population

**Figure 1:**
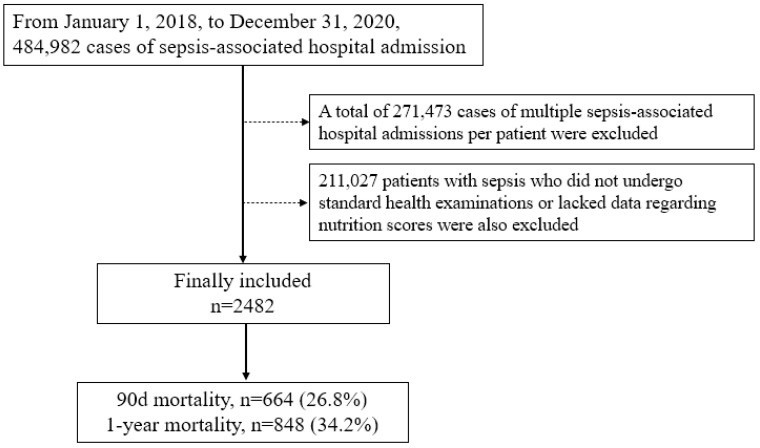
Flow chart depicting the patient selection process.

**Figure 2:**
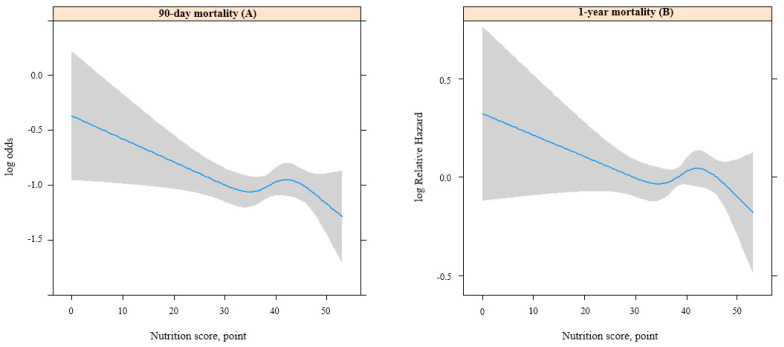
Log odds of 90-day mortality and log relative hazard of 1-year mortality in restricted cubic splines.

Figure 1 shows the selection process of patients with sepsis for this study. From 1 January 2018 to 31 December 2020, 484,982 sepsis-associated hospital admission cases were initially screened. A total of 271,473 patients with multiple (two or more) sepsis-associated hospital admissions were excluded, and 211,027 patients with sepsis who did not undergo standard health examinations before hospital admission or lacked data regarding nutrition scores were also excluded. Thus, 2482 patients with sepsis were included in this study. The 90-day and 1-year mortality rates in patients with sepsis were 26.8% (664/2482) and 34.2% (848/2482), respectively. The clinicopathological characteristics of patients with sepsis are presented in Table 2. The median value of age was 63.0 years old (IQR = 61.0–72.0, range = 40.0–73.0), and the proportion of female patients was 46.0% (1141/2482). A total of 23.8% (590/2482) of patients with sepsis were admitted to the ICU. Figure 2A,B show the log odds of 90-day mortality and log relative hazard of 1-year mortality according to the nutrition scores using RCSs.

### 3.2. Survival Analyses

Table 3 shows the results of survival analyses. In the covariate-adjusted multivariable logistic regression model, a 1-point increase in the nutrition score was associated with a decrease in 90-day mortality in patients with sepsis (OR: 0.98, 95% CI: 0.97, 0.98; *p* = 0.025). In the covariate-adjusted multivariable Cox regression model, a 1-point increase in the nutrition score was associated with a decrease in 1-year mortality in patients with sepsis (H.R.: 0.99, 95% CI: 0.98, 0.99; *p* = 0.035). All other ORs and HRs with 95% CIs of covariates in the multivariable models are presented in Tables S2 and S3.

### 3.3. Subgroup Analyses Based on BMI

Table 4 shows the results of the subgroup analyses based on the BMI group. A 1-point increase in the nutrition score was associated with decreased 90-day and 1-year mortality in the underweight group (<18.5 kg/m^2^) (90-day mortality, OR: 0.95, 95% CI: 0.89, 0.98; *p* = 0.023; 1-year mortality: H.R.: 0.97, 95% CI: 0.95, 0.99; *p* = 0.040). However, the nutrition score was not associated with either 90-day or 1-year mortality for the normal (18.5–24.9 kg/m^2^), overweight (25.0–29.9 kg/m^2^), and obese (>30.0 kg/m^2^) BMI groups.

## 4. Discussion

Patients who were diagnosed with sepsis were subjected to a population-based cohort research, which revealed that a higher antecedent nutrition status was associated with lower mortality rates at 90 days and 1 year. In addition, this connection was significant in patients with a BMI of 18.5 kg/m^2^ or below. According to the findings of this study, one of the most important factors in determining whether or not a patient will survive sepsis is their nutritional state.

Malnutrition is an important predictor of health outcomes in the elderly population, and the impact of malnutrition on hospitalization outcomes in older persons treated with sepsis is an important issue [14]. Abugroun et al. reported that malnutrition is an independent predictor of poor hospitalization outcomes in the senior population with sepsis [9]. Moreover, Adejumo et al. also reported that protein-energy malnutrition is a risk factor for sepsis and has been linked to poorer outcomes in sepsis patients [15]. We assessed the prior nutritional status using the Nutritional Lifestyle Assessment Tool in this study as a novel tool.

Because we measured prior nutritional status with the Nutritional Lifestyle Assessment Tool in South Korea, our findings contribute something new to the scientific community. As can be seen in Table S1, the questionnaire is composed of straightforward inquiries that investigate individuals’ eating behaviors and the extent to which they receive an acceptable amount of essential nutrients. As a result, this questionnaire could be utilized in different nations to evaluate the nutritional health of individuals who are afflicted with sepsis or any number of other disorders.

Furthermore, the Nutritional Lifestyle Assessment Tool in South Korea uses a simple questionnaire to assess an appropriate and balanced diet. In other words, it is possible to assess whether the main nutrient intake, including micronutrients, is enough. Micronutrient insufficiency is considered to be a major component in the poor prognosis of sepsis patients [16]. A prospective observational study in an infectious disease clinic reported that there are novel correlations between the levels of micronutrients, the type of infection, and the negative clinical effects [17].

Because nutrition is so intimately linked to immune function, the nutritional state of a patient with sepsis may be an extremely important and influential component that can be altered [18,19]. Proper nutrition in conjunction with an adequate intake of dietary supplements can have an effect on both the innate and adaptive immune systems, as well as on their ability to function normally [18,19]. Zinc, iron, copper, selenium, and the vitamins A, B12, B6, C, and E, all of which can be supplemented, are critical elements that contribute to the maintenance of the immune cells’ capacity to defend the body [20]. Therefore, immune-modulating nutrition may be of critical importance for lowering the risk of infectious complications and minimizing the length of stay for patients undergoing surgery for gastrointestinal cancer [21].

By inhibiting the immune system and causing persistent inflammation, sepsis can change both the innate and adaptive immune responses [22]. A host response that is dysregulated is characterized by unbalanced immune suppression and hyperinflammation, both of which have the potential to lead to organ dysfunction and increased mortality associated with sepsis [23]. Therefore, providing patients who have sepsis with nutritional support is quite important. Early enteral nutritional assistance has been found in a previous cohort research study to have the potential to prevent overactive immune responses, as well as minimize the length of stays in the intensive care unit (ICU), hospitalization, and the duration of mechanical breathing in patients who have sepsis. [24]. However, there is not enough research available on the connection between a history of malnutrition and survival outcomes in people who have sepsis. Recent research conducted by Abugroun and colleagues demonstrates that a history of malnutrition is a significant factor in poor prognosis during hospitalization among elderly patients who have been diagnosed with sepsis [9].

Recent research by Gao and colleagues suggests that nutritional risk screening (NRS) scores could be used to effectively stratify patients who are suffering from sepsis [25]. The NRS scores include BMI < 20.5 kg/m^2^, weight loss within three months, reduced dietary intake, or ICU admission [26]. However, as can be seen in Table S1, the Nutritional Lifestyle Assessment Tool that is used in South Korea consists of 11 questions that are centered on the subject of food consumption and eating patterns. Consuming healthy foods and maintaining healthy eating habits has been linked to increased immune function [27,28]. As a result, the Nutritional Lifestyle Assessment Tool is able to evaluate a person’s nutritional status in terms of a balanced diet and good eating habits, but it does not take BMI into account.

The findings of the subgroup analyses with regard to BMI scores before hospital admission in individuals with sepsis are essential. Patients with sepsis who were underweight (defined as having a BMI score of 18.5 or less kg/m^2^) were the only ones for whom an improved preceding nutritional status was significantly associated with improved survival outcomes. Previous research found that higher body mass index (BMI) values were independently related with improved survival outcomes in patients who were diagnosed with sepsis [29,30]. Patients in a Japanese cohort who had a BMI score of less than 18.5 kg/m^2^ and severe sepsis had a considerably increased risk of dying within the first 28 days, according to a study that was published by Oami et al. [31]. Our findings imply that appropriate dietary intake is a controllable and favorable factor for decreased mortality in patients with BMI scores lower than 18.5 kg/m^2^ and sepsis. This conclusion is supported by the fact that adequate dietary intake was a factor in our study.

We can use these data to identify sepsis patients who may be at risk of death owing to malnutrition at the time of hospitalization. As a result, medical staff can consider providing nutritional support such as enteral nutrition or parenteral nutrition to these high-risk malnutrition patients earlier. Nutritional support for critically ill patients remains controversial and requires special attention in clinical practice. Auiwattanakul et al. reported that enteral nutrition or parenteral nutrition were associated with improved 28-day mortality in patients with sepsis treated in surgical ICUs [10]. Another cohort study by Cha et al. reported that early nutritional supply with EN and/or PN was associated with improved survival outcomes in patients with sepsis or septic shock [32]. Insufficient nutritional support is associated with immune dysfunction in patients with sepsis [33]. In this background, our findings can be used to evaluate patients with sepsis who need nutritional support.

Our study had some limitations. First, it excluded patients with sepsis who did not undergo standard health examination before hospital admission or lacked data regarding nutrition scores; a large sample size was excluded, which might have affected the results. Second, the severity of sepsis was not evaluated in detail using disease scoring systems such as the Acute Physiology and Chronic Health Evaluation II scores or Sequential Organ Failure Assessment scores. Third, as the nutrition scores were evaluated using a survey, some bias might be at play, such as response or selection bias [34]. Fourth, as the number of patients from rural areas was large and surgery-associated sepsis hospital admission appears high, it could lead to selection bias in our cohort study. Fifth, we only used the ICD-10 code of sepsis; the source of infection was not considered in this study. As the etiology of sepsis could affect the relationship between prior nutritional status and survival outcomes, future study is needed to confirm this issue considering the etiology of sepsis. Moreover, the immune status of individual patients could not be evaluated, which could be a limitation, too. Sixth, residual or unmeasured confounders may have affected the study’s results. Lastly, our study used a simple questionnaire (Nutritional Lifestyle Assessment Tool) to test nutritional status, which the general public could easily answer. This has the advantage of being very simple, quick, and easy for anyone to execute, especially people with little education. As a result, it is feasible to check the nutritional condition of all patients in advance in a simple and quick manner. However, nutritionally important factors such as sugar intake, refined sweets, total calorie intake, and consumption of natural antioxidants, spices, and adjuvants were not reflected. Moreover, cultural differences in eating habits might vary from country to country or area to area regarding nutrition. For example, there are big differences in dietary habits between Asian and Western countries; in Asia, there is a good intake of healthy foods, e.g., sea foods and specific spices, and the consumption is more rational and calorie-limited, while in the Western world, there is an excess of industrialized and refined foods that has resulted in an explosion of obesity and nutrition-related disorders in recent times. Therefore, our results should be interpreted carefully. When this questionnaire (Nutritional Lifestyle Assessment Tool) is utilized in other nations, it must be modified to reflect the features of the region and country.

## 5. Conclusions

In this population-based cohort study, a better nutritional status before hospital admission was associated with improved 90-day and 1-year mortality rates among patients with sepsis. This association was more evident in underweight patients (BMI < 18.5 kg/m^2^). Our results suggest that adequate dietary intake and healthy eating habits might be a protective factor against mortality among patients with sepsis.

## Figures and Tables

**Table 1:** Nutritional Lifestyle Assessment Tool.

Name (Chart Number):
1. I drink more than one cup (200 mL) of milk, calcium-fortified soymilk, or any other dairy products (yogurt, etc.) every day.
(1) always (5 points) (2) regularly (3 points) (3) almost never or never (1 point)
2. I eat a dish consisting of meat, fish, eggs, soy, tofu, etc., more than three times a day.
(1) always (5 points) (2) regularly (3 points) (3) almost never or never (1 point)
3. I eat vegetables excluding kimchi at every meal.
(1) always (5 points) (2) regularly (3 points) (3) almost never or never (1 point)
4. I eat fruit (one whole piece) every day (including grounded types)
(1) always (5 points) (2) regularly (3 points) (3) almost never or never (1 point)
5. How often do you eat fried or stir-fried food?
(1) always (5 points) (2) regularly (3 points) (3) almost never or never (1 point)
6. How often do you eat foods high in cholesterol (pork belly, egg yolk, squid, etc.)?
(1) always (5 points) (2) regularly (3 points) (3) almost never or never (1 point)
7. I eat at least one type of ice cream, cake, snacks, or drinks (instant coffee premixed with non-dairy cream and sugar, coke, sikhye (sweet rice drink)) every day.
(1) always (5 points) (2) regularly (3 points) (3) almost never or never (1 point)
8. I eat salted seafood, pickled vegetables, or salted dry fish every day.
(1) always (5 points) (2) regularly (3 points) (3) almost never or never (1 point)
9. I have a meal every day at a fixed time.
(1) always (5 points) (2) regularly (3 points) (3) almost never or never (1 point)
10. How many types of food do you usually eat per day out of a total of 5 types including grains (rice, breads), meat/fish/eggs/beans, vegetables, fruits, and dairy products?
(1) 5 types (5 points) (2) 4 types (3 points) (3) fewer than or equal to 3 types (1 point)
11. How often do you eat out (excluding meals provided at work)?
(1) More than 5 times a week (1 point) (2) 2–4 times a week (3 points) (3) less than or equal to once a week (5 points)

**Table 2:** Clinicopathological characteristics of patients with sepsis.

Variable	Median [IQR, Range] or N (%)
Age, year	63.0 [61–72, 40–73]
Female sex	1141 (46.0)
Main diagnosis of sepsis	1655 (66.7)
Having a job	1150 (46.3)
Residence at sepsis	
Urban area	750 (30.2)
Rural area	1732 (69.8)
BMI, kg/m^2^	24.3 (3.9)
<18.5 (underweight)	134 (5.4)
18.5–24.9 (normal)	1368 (55.1)
25.0–29.9 (overweight)	800 (32.2)
>30.0 (obese)	180 (7.3)
Nutrition score before hospital admission	38.6 (7.7)
CCI, point	3.2 (3.1)
Disability at hospital admission	
Mild to moderate	148 (5.8)
Severe	111 (4.5)
ICU admission	590 (23.8)
Ventilator support	313 (12.6)
ECMO support	16 (0.6)
CRRT use	113 (4.6)
Type of hospital	
General hospital	1927 (77.6)
Hospital	164 (6.6)
Long-term facility care hospital	391 (15.8)
IM department	1655 (66.7)
Surgery-associated hospital admission	884 (35.6)
LOS, day	11.6 (11.3)
Total cost for hospitalization in USD	5215.3 (8855.1)
Year	
2018	615 (24.8)
2019	829 (33.4)
2020	1038 (41.8)

BMI, body mass index; CCI, Charlson comorbidity index; ICU, intensive care unit; ECMO, extracorporeal membrane oxygenation; CRRT, continuous renal replacement therapy; IM, internal medicine; LOS, length of hospital stays; USD, United States Dollars.

**Table 3:** Survival analyses according to nutrition score.

Variable	OR (95% CI) or HR (95% CI)	*p*-Value
Unadjusted Analysis for 90-Day Mortality		
Nutrition score, 1 point increase	0.99 (0.98, 1.00)	0.059
Unadjusted analysis for 1-year mortality		
Nutrition score, 1 point increase	0.99 (0.98, 1.00)	0.062
Covariate-adjusted model for 90-day mortality (model 1)		
Nutrition score, 1 point increase	0.98 (0.97, 0.98)	0.025
Covariate-adjusted model for 1-year mortality (model 2)		
Nutrition score, 1 point increase	0.99 (0.98, 0.99)	0.035

Hosmer–Lemeshow statistics of model 1: Chi-square, 7.34, Df = 8, *p* = 0.500. OR, odds ratio; HR, hazard ratio.

**Table 4:** Subgroup analyses according to BMI group.

Variable	OR (95% CI) or HR (95% CI)	*p*-Value
90-Day Mortality		
BMI < 18.5 kg/m^2^		
Nutrition score, 1 point increase	0.95 (0.89, 0.98)	0.023
BMI: 18.5–24.9 kg/m^2^		
Nutrition score, 1 point increase	0.98 (0.97, 1.00)	0.065
BMI: 25.0–29.9 kg/m^2^		
Nutrition score, 1 point increase	0.99 (0.96, 1.02)	0.514
BMI > 30.0 kg/m^2^		
Nutrition score, 1 point increase	1.01 (0.96, 1.07)	0.667
1-year mortality		
BMI < 18.5 kg/m^2^		
Nutrition score, 1 point increase	0.97 (0.95, 0.99)	0.040
BMI: 18.5–24.9 kg/m^2^		
Nutrition score, 1 point increase	0.99 (0.98, 1.01)	0.298
BMI: 25.0–29.9 kg/m^2^		
Nutrition score, 1 point increase	0.98 (0.97, 1.01)	0.199
BMI > 30.0 kg/m^2^		
Nutrition score, 1 point increase	1.01 (0.97, 1.06)	0.660

BMI, body mass index; OR, odds ratio; CI, confidence interval; HR, hazard ratio.

## Data Availability

Data are contained within the article.

## Supplementary Materials

The following supporting information can be downloaded at: https://www.mdpi.com/article/10.3390/nu15245040/s1, Table S1: The ICD-10 codes are used by comorbidity to compute the CCI; Table S2: All ORs with 95% CIs in multivariable model 1; Table S3: All HRs with 95% CIs in multivariable model 2.
