# Supplementary material for: Prior Evaluation of Nutritional Status and Mortality in Patients with Sepsis in South Korea
Source: Nutrients. 2023 Dec 8;15(24):5040. doi: 10.3390/nu15245040 (PMC10745302; doi:10.3390/nu15245040)
Supplement: Supplementary file 1 [file nutrients-15-05040-s001.zip › nutrients-2722470-supplementary.pdf]

Table S1. The ICD-10 codes used by comorbidity to compute the Charlson comorbidity index.

**The ICD-10 codes used by comorbidity to compute the Charlson comorbidity index are:**	
○	Myocardial infarction: I21.x, I22.x, I25.2
○	Congestive heart failure: I09.9, I11.0, I13.0, I13.2, I25.5, I42.0, I42.5 - I42.9, I43.x, I50.x, P29.0
○	Peripheral vascular disease: I70.x, I71.x, I73.1, I73.8, I73.9, I77.1, I79.0, I79.2, K55.1, K55.8, K55.9, Z95.8, Z95.9
○	Cerebrovascular disease: G45.x, G46.x, H34.0, I60.x - I69.x
○	Dementia: F00.x - F03.x, F05.1, G30.x, G31.1
○	Chronic pulmonary disease: I27.8, I27.9, J40.x - J47.x, J60.x - J67.x, J68.4, J70.1, J70.3
○	Rheumatic disease: M05.x, M06.x, M31.5, M32.x - M34.x, M35.1, M35.3, M36.0
○	Peptic ulcer disease: K25.x - K28.x
○	Mild liver disease: B18.x, K70.0 - K70.3, K70.9, K71.3 - K71.5, K71.7, K73.x, K74.x, K76.0, K76.2 - K76.4, K76.8, K76.9, Z94.4
○	Diabetes without chronic complication: E10.0, E10.1, E10.6, E10.8, E10.9, E11.0, E11.1, E11.6, E11.8, E11.9, E12.0, E12.1, E12.6, E12.8, E12.9, E13.0, E13.1, E13.6, E13.8, E13.9, E14.0, E14.1, E14.6, E14.8, E14.9
○	Diabetes with chronic complication: E10.2 - E10.5, E10.7, E11.2 - E11.5, E11.7, E12.2 - E12.5, E12.7, E13.2 - E13.5, E13.7, E14.2 - E14.5, E14.7
○	Hemiplegia or paraplegia: G04.1, G11.4, G80.1, G80.2, G81.x, G82.x, G83.0 - G83.4, G83.9
○	Renal disease: I12.0, I13.1, N03.2 - N03.7, N05.2 - N05.7, N18.x, N19.x, N25.0, Z49.0 - Z49.2, Z94.0, Z99.2
○	Any malignancy, including lymphoma and leukaemia, except malignant neoplasm of skin: C00.x - C26.x, C30.x - C34.x, C37.x - C41.x, C43.x, C45.x - C58.x, C60.x - C76.x, C81.x - C85.x, C88.x, C90.x - C97.x
○	Moderate or severe liver disease: I85.0, I85.9, I86.4, I98.2, K70.4, K71.1, K72.1, K72.9, K76.5, K76.6, K76.7
○	Metastatic solid tumour: C77.x - C80.x
○	AIDS/HIV: B20.x - B22.x, B24.x

Table S2. All ORs with 95% CIs in multivariable model 1.

**Variable**	**OR (95% CI)**	***p*-Value**
Age, year	1.04 (1.02, 1.05)	<0.001
Female sex	0.61 (0.49, 0.76)	<0.001
Main diagnosis of sepsis	0.75 (0.57, 0.97)	0.027
Having a job	0.75 (0.59, 0.94)	0.012
Residence at sepsis		
Urban area	1	
Rural area	1.10 (0.86, 1.40)	0.468
BMI		
<18.5 (underweight)	1.56 (1.01, 2.43)	0.048
18.5–24.9 (normal)	1	
25.0–29.9 (overweight)	0.74 (0.58, 0.95)	0.016
>30.0 (obese)	0.92 (0.59, 1.42)	0.6978
CCI, point	1.05 (1.03, 1.07)	<0.001
Disability at hospital admission		
Mild to moderate	0.68 (0.40, 1.15)	0.149
Severe	0.73 (0.42, 1.27)	0.270
ICU admission	0.92 (0.66, 1.30)	0.645
Ventilator support	11.38 (7.73, 16.74)	<0.001
ECMO support	1.20 (0.22, 6.61)	0.838
CRRT support	6.45 (3.26, 12.77)	<0.001
Type of hospital		
General hospital	1	
Hospital	1.10 (0.69, 1.76)	0.686
Long-term facility care hospital	4.83 (3.42, 6.80)	<0.001
IM department	1.90 (1.48, 2.44)	<0.001
Surgery associated hospital admission	1.46 (1.11, 1.91)	0.007
Year		
2018	1	
2019	2.56 (1.84, 3.55)	<0.001
2020	2.09 (1.50, 2.91)	<0.001

OR, odds ratio; CI, confidence interval; BMI, body mass index; CCI, Charlson comorbidity index; ICU, intensive care unit; ECMO, extracorporeal membrane oxygenation; CRRT, continuous renal replacement therapy; IM, internal medicine.

Table S3. All HRs with 95% CIs in multivariable model 2.

Variable	HR (95% CI)	*p*-Value
Age, year	1.03 (1.02, 1.03)	<0.001
Female sex	0.71 (0.62, 0.83)	<0.001
Main diagnosis of sepsis	0.75 (0.64, 0.89)	0.001
Having a job	0.83 (0.72, 0.96)	0.013
Residence at sepsis		
Urban area	1	
Rural area	1.01 (0.87, 1.18)	0.877
BMI		
<18.5 (underweight)	1.38 (1.05, 1.81)	0.020
18.5–24.9 (normal)	1	
25.0–29.9 (overweight)	0.74 (0.63, 0.87)	<0.001
>30.0 (obese)	0.79 (0.58, 1.06)	0.112
CCI, point	1.03 (1.02, 1.04)	<0.001
Disability at hospital admission		
Mild to moderate	0.74 (0.53, 1.05)	0.075
Severe	0.85 (0.60, 1.21)	0.373
ICU admission	0.89 (0.71, 1.13)	0.339
Ventilator support	4.54 (3.64, 5.67)	<0.001
ECMO support	0.65 (0.37, 1.15)	0.136
CRRT support	2.45 (1.88, 3.19)	<0.001
Type of hospital		
General hospital	1	
Hospital	0.96 (0.68, 1.36)	0.816
Long-term facility care hospital	2.99 (2.39, 3.74)	<0.001
IM department	1.37 (1.18, 1.60)	<0.001
Surgery associated hospital admission	1.29 (1.07, 1.55)	0.008
Year		
2018	1	
2019	1.87 (1.51, 2.32)	<0.001
2020	1.71 (1.37, 2.13)	<0.001

HR, hazard ratio; CI, confidence interval; BMI, body mass index; CCI, Charlson comorbidity index; ICU, intensive care unit; ECMO, extracorporeal membrane oxygenation; CRRT, continuous renal replacement therapy; IM, internal medicine.
